# Leucine-rich repeat kinase 2 at a glance

**DOI:** 10.1242/jcs.259724

**Published:** 2023-09-12

**Authors:** Christiane Zhu, Susanne Herbst, Patrick A. Lewis

**Affiliations:** ^1^ Department of Comparative Biomedical Sciences, Royal Veterinary College, University of London, London NW1 0TU, UK; ^2^ Department of Neurodegenerative diseases, UCL Queen Square Institute of Neurology, University College London, London WC1N 3BG, UK; ^3^ Aligning Science Across Parkinson's (ASAP) Collaborative Research Network, Chevy Chase, MD 20815, USA

**Keywords:** Leucine-rich repeat kinase 2, GTPase, Kinase, Structure, Function, Cellular processes, Parkinson's disease

## Abstract

Leucine-rich repeat kinase 2 (LRRK2) is a multidomain scaffolding protein with dual guanosine triphosphatase (GTPase) and kinase enzymatic activities, providing this protein with the capacity to regulate a multitude of signalling pathways and act as a key mediator of diverse cellular processes. Much of the interest in LRRK2 derives from mutations in the *LRRK2* gene being the most common genetic cause of Parkinson's disease, and from the association of the *LRRK2* locus with a number of other human diseases, including inflammatory bowel disease. Therefore, the LRRK2 research field has focused on the link between LRRK2 and pathology, with the aim of uncovering the underlying mechanisms and, ultimately, finding novel therapies and treatments to combat them. From the biochemical and cellular functions of LRRK2, to its relevance to distinct disease mechanisms, this Cell Science at a Glance article and the accompanying poster deliver a snapshot of our current understanding of LRRK2 function, dysfunction and links to disease.

## Teaser

**Summary:** We provide a snapshot of the current understanding of the biology and pathological roles of leucine-rich repeat kinase 2.

## Introduction

Leucine-rich repeat kinase 2 (LRRK2) is a multidomain, multifunctional scaffolding protein and enzyme (Marchand et al., 2020). It possesses several protein–protein interaction (PPI) domains, in addition to a catalytic core encompassing a ROCO guanosine triphosphatase (GTPase) supradomain, which consists of a Ras of complex proteins (ROC) domain and a C-terminal of ROC (COR) domain. These are positioned in tandem and are followed by a serine/threonine kinase domain (see poster) (Berwick et al., 2019), establishing LRRK2 as one of only three dual-activity GTPase–kinase proteins in the human proteome – the other two [LRRK1 and death-associated protein kinase 1 (DAPK1)] are likewise members of the ROCO protein superfamily. Importantly, the non-catalytic PPI domains allow LRRK2 to act as a signalling scaffold protein, bringing together distinct binding partners to mediate cell signal transduction cascades and pathways (Wallings et al., 2015).

This multifunctional aspect of LRRK2 contributes to its involvement in different human diseases, including cancer (Ermine et al., 2022), leprosy (Zhang et al., 2009), Crohn's disease (Franke et al., 2010) and other inflammatory diseases (Wang et al., 2018). However, LRRK2 remains primarily associated with Parkinson's disease (PD), a progressive neurological disorder characterised by prominent motor symptoms resulting from the selective loss of dopaminergic neurons in the substantia nigra (Bloem et al., 2021). Although most PD cases are idiopathic, there are familial cases – including autosomal-dominant disease caused by coding mutations in the *LRRK2* gene (Zimprich et al., 2004). Understanding the switch from physiological function to a pathological role for LRRK2, and thereby gaining mechanistic insights into PD pathogenesis and neurodegeneration, has therefore been a primary objective for the LRRK2 field (Cookson, 2010; Usmani et al., 2021). Substantial efforts have also been directed towards developing strategies to target LRRK2 (Kluss et al., 2022). Thus, LRRK2 is of significant interest in PD and other diseases; however, a greater understanding of its many functions is required before we can fully comprehend the consequences of dysfunction. This Cell Science at a Glance article and the accompanying poster summarise our current understanding of LRRK2 in health and disease.

**Figure:**
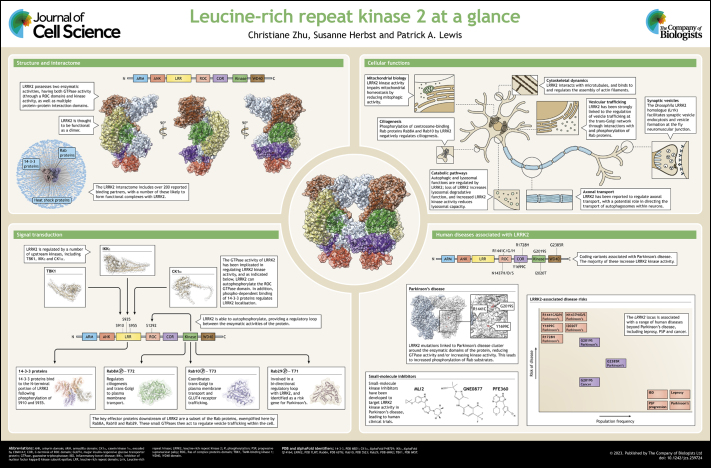
See Supplementary information for a high-resolution version of the poster.

## LRRK2 – structure and function

### The domain architecture of LRRK2

LRRK2 possesses a complex domain organisation with seven domains, including five non-catalytic PPI domains and two enzymatic domains (Civiero et al., 2014). The N-terminal PPI domains comprise an ankyrin (Ank) domain, an armadillo (Arm) domain and the eponymous leucine-rich repeat (LRR) domain, with a WD40 domain located at C terminus of the protein (Marín, 2008; Piccoli et al., 2014) (see poster). The Arm domain drives interaction with Rab GTPases, which mediate LRRK2 recruitment to intracellular membranes (Steger et al., 2016; Vides et al., 2022). The functions of the remaining PPI domains are less clear; they most likely play a crucial part in the scaffold function of LRRK2, allowing diverse molecular partners to bind to LRRK2 and influence signal transduction cascades (Tomkins et al., 2018).

The catalytic core of LRRK2 consists of a ROC–COR–kinase arrangement, which can be subdivided into the tandem ROC–COR supradomain and the kinase domain. The COR domain is sandwiched between the two enzymatic domains of LRRK2 and has been identified as the primary site mediating LRRK2 dimerisation (Myasnikov et al., 2021). The presence of the tandem ROC–COR supradomain defines LRRK2 as a member of the ROCO protein superfamily (Civiero et al., 2014; Humphries et al., 2015). The ROCO proteins are a heterogenous group of multidomain signalling proteins first described in the amoeba *Dictyostelium discoidium* and are distinguished by a highly versatile modular domain organisation, with the ROC domain thought to act as a molecular switch analogous to the small GTPases (typified by Ras, after which the ROCO proteins are named) (Bosgraaf and Van Haastert, 2003). LRRK2 can both bind and hydrolyse GTP, and it is therefore an active GTPase (Guo et al., 2007; Lewis et al., 2007). Despite the homology to small GTPases, LRRK2 is an unconventional GTPase, partly because of the lack of confirmation of a guanine-nucleotide-exchange factor (GEF), and it has been proposed that dimerisation is a key driver of GTP hydrolysis (Gasper et al., 2009).

The kinase domain of LRRK2 is most closely related to the receptor-interacting protein kinase (RIPK) family of serine/threonine kinases, with LRRK2 designated as RIPK7 (Meylan and Tschopp, 2005; Zhang et al., 2010; Humphries et al., 2015). LRRK2 has been reported to phosphorylate a wide range of substrates – most notably and reproducibly a subset of Rab GTPases (Steger et al., 2016) – as well as displaying autophosphorylation (Sheng et al., 2012). Similar to the GTPase activity of LRRK2, there is evidence that dimerisation plays an important role in the activation and regulation of LRRK2 kinase activity (Greggio et al., 2008; Sen et al., 2009).

Congruent with a model in which the function of the LRRK2 GTPase domain regulates kinase activity, the ROC–COR domain undergoes major structural changes when LRRK2 is in a kinase-active conformation (Myasnikov et al., 2021). GTP binding is a prerequisite for LRRK2 kinase function, and an increase in GTPase function might reduce kinase activity. Conversely, LRRK2 pathogenic mutations in the ROC domain have been suggested to increase kinase activity by decreasing GTPase activity (Biosa et al., 2012; Ito et al., 2007; Lewis et al., 2007; Nixon-Abell et al., 2016; Wang et al., 2021). Interestingly, the ROC domain harbours several autophosphorylation sites that potentially act to regulate GTPase activity, although the mechanism by which this might occur is unclear (Greggio et al., 2009; Ito et al., 2007).

### The tertiary and quaternary structure of LRRK2

The large size of LRRK2 (in excess of 250 kDa) makes it a challenging candidate for structural studies, with initial efforts having focused on the elucidation of structures for individual domains in isolation (Deng et al., 2008; Gilsbach et al., 2012). The past several years, however, have witnessed a revolution in our understanding of LRRK2 structure at an Ångström resolution – resulting from the application of cryogenic electron microscopy – with the publication of multiple structures for large fragments of LRRK2 (Deniston et al., 2020; Watanabe et al., 2020) and the full-length protein (Myasnikov et al., 2021) (see poster). This has revealed a diverse range of potential conformations, ranging from monomeric structures, through to dimers, trimers and tetramers. How these conformations relate to the cellular function of LRRK2 is currently unclear and is the subject of much ongoing research (Herbst and Lewis, 2021).

### LRRK2 posttranslational modifications

Possessing several PPI domains allows LRRK2 to interact with numerous proteins and to regulate diverse signalling pathways. LRRK2 itself is the target of posttranslational modifications, directing the downstream activity of the protein. LRRK2 possesses multiple phosphorylation sites, which are subjected to autophosphorylation (Kamikawaji et al., 2009) or phosphorylation by other kinases (see poster). Although the physiological kinases and phosphatases that act on LRRK2 are yet to be fully characterised, a number of candidates have been reported, including casein kinase 1α (CK1α, encoded by *CSNK1A1*) (Chia et al., 2014; De Wit et al., 2019), TANK-binding kinase 1 (TBK1) (Hermanson et al., 2012), inhibitor of nuclear factor kappa-B kinase subunit epsilon (IKKε) (Dzamko et al., 2012; Hermanson et al., 2012) and protein kinase A (PKA) (Greggio et al., 2017; Russo, 2019). With regard to dephosphorylation, protein phosphatase 1 (PP1) has been suggested (Lobbestael et al., 2013).

Although posttranslational modification mapping studies have revealed numerous potential phosphorylation sites for LRRK2, only a few of these sites have been robustly validated in multiple independent studies, including sites S910, S935, S955 and S973 (Li et al., 2011), as well as autophosphorylation at S1292 (Sheng et al., 2012), T1491 and T2483 (Kamikawaji, et al., 2009; Gloeckner et al., 2010). Of note, autophosphorylation sites have been discovered *in vitro* in both the kinase and GTPase domains of LRRK2, suggesting an interplay between the kinase and GTPase activities (Greggio et al., 2009). Maintaining the balance between phosphorylation and dephosphorylation of LRKK2 might be fundamental for its normal function, cellular distribution and regulation of biological processes. This is exemplified by the phospho-dependent binding of the family of 14-3-3 adapter proteins to S910 and S935 of LRRK2 (De Wit et al., 2018). It has been suggested that interactions of 14-3-3 proteins with LRRK2 could regulate its cytoplasmic localisation and stabilisation (Nichols et al., 2010), as mutations of *LRRK2* affecting S910 and S935 lead to the accumulation of LRRK2 within cytoplasmic pools containing misfolded, unstable LRRK2 protein (Nichols et al., 2010; Dzamko et al., 2010).

Beyond phosphorylation, LRRK2 is also subject to ubiquitylation on various leucine residues. The inhibition of LRRK2 kinase activity has been reported to increase LRRK2 dephosphorylation whilst reducing the stability of the protein downstream and increasing its ubiquitylation and subsequent degradation (Zhao et al., 2015). Comparably, LRRK2 ubiquitylation has been observed to increase when GTP binding is inhibited (Thomas et al., 2020). It is of note that the posttranslational modifications of LRRK2 beyond phosphorylation remain understudied.

### LRRK2 substrates

In 2016, a seminal phosphoproteomics study identified a subset of Rab GTPases, including Rab3A–D, Rab8A, Rab8B, Rab10, Rab12, Rab29, Rab35 and Rab43 as bona fide LRRK2 substrates (Steger et al., 2016) (see poster). Rab GTPases play a fundamental role in membrane and vesicle trafficking, and are phosphorylated by LRRK2 in their membrane-bound state (Pfeffer, 2018). LRRK2-dependent phosphorylation of Rab GTPases stabilises their membrane-bound form and alters the profile of effector proteins (Usmani et al., 2021), with consequences for Rab activity impacting on ciliogenesis and lysosomal homeostasis (Lara Ordónez et al., 2019; Steger et al., 2017). A number of other proteins have been reported to be phosphorylated by LRRK2, including (but not limited to) 14-3-3 proteins (Rudenko and Cookson, 2010), p53 (also known as TP53) (Ho et al., 2015) and p62 (also known as SQSTM1) (Kalogeropulou et al., 2018). Extensive physiological validation of these substrates, however, is lacking.

### Cellular functions of LRRK2

The multidomain architecture and dual enzymatic activities of LRRK2 lend themselves to an association with a myriad of processes within the cell. Through interactions with diverse binding partners (Manzoni et al., 2015), LRRK2 acts as a signalling hub, playing a role in growth factor, immune, survival and death-receptor signalling pathways, among which the nuclear factor of activated T cells (NFAT), Wnt, Akt, mammalian target of rapamycin (mTOR), ERK and Toll-like receptor (TLR) pathways can be mentioned (Sancho et al., 2009; Dzamko et al., 2012; Reinhardt et al., 2013; Chuang et al., 2014; Berwick et al., 2017; Harvey and Outeiro, 2019). A key area of interest regarding LRRK2 is its relationship with different vesicular trafficking events (Sanna et al., 2012), particularly its regulatory role in catabolic pathways, including the endolysosomal (Kuwahara and Iwatsubo, 2020) and autophagic systems (Manzoni, 2012; Manzoni and Lewis, 2017). LRRK2 has been proposed to regulate vesicle trafficking in these pathways through interactions with Rab proteins (Bae and Lee, 2020), including phosphorylation of Rab8A and Rab10 (Steger et al., 2016).

LRRK2 is involved in regulating clathrin-mediated endocytosis of mammalian synaptic vesicles (Arranz et al., 2015; Heaton et al., 2020), and there is evidence that this is conserved through evolution. As a regulator of Endophilin A, a key protein involved in synaptic vesicle endocytosis, the *Drosophila melanogaster* LRRK2 homologue Lrrk facilitates vesicle formation at neuromuscular junction synapses (Matta et al., 2012; Inoshita et al., 2017). Additionally, *LRRK2* mutations result in dysregulation of the endolysosomal system, which in turn has been speculated to take part in disease mechanisms related to PD (for a review, see Erb and Moore, 2020). For instance, *LRRK2*-knockout animals present altered morphology and function of lysosomes (Fuji et al., 2015; Kuwahara et al., 2016). Knockout of *LRRK2* has repeatedly been shown to result in increased lysosomal degradative function, and LRRK2 pathogenic mutations that increase kinase activity decrease the degradative capacity of lysosomes (Cogo et al., 2020). Altered lysosomal function because of altered LRRK2 kinase activity can also be observed in carriers of mutant *LRRK2*; independently of PD status, they show an increase in the lysosomal lipid bis(monoacylglycero)phosphate (BMP) in urine, which is reversed by LRRK2 kinase inhibition (Jennings et al., 2022; Merchant et al., 2023). Urinary BMP has thus emerged as a relevant biomarker of lysosomal function in individuals with PD who harbour *LRRK2* mutations.

LRRK2 also regulates ciliogenesis; upon phosphorylation by LRRK2, membrane-bound Rab8A and Rab10 accumulate at centrosomes (Babbey et al., 2010), where they contribute to a number of deficits in ciliogenesis and centrosomal cohesion (Lara Ordónez et al., 2019; Madero-Pérez et al., 2018). Centrosomes organise the cellular cytoskeleton, thereby playing a central role in cell division, and form the building blocks of cilia, which are important signalling hubs. In line with this, centrosome cohesion defects can be observed in induced pluripotent stem cells carrying *LRRK2* pathogenic mutations (Fdez et al., 2022; Lara Ordóñez et al., 2022) and *LRRK2*-mutant knock-in mice display reduced primary ciliogenesis (Steger et al., 2017). In particular, LRRK2-mediated alterations to ciliogenesis have been validated in striatal cholinergic neurons of *LRRK2* R1441C homozygous knock-in mice (Dhekne et al., 2018; Khan et al., 2021) and *LRRK2* G2019S knock-in mouse models (Khan et al., 2021), as well as in primary astrocytes from *LRRK2* G2019S homozygous knock-in mice (Lara Ordónez et al., 2019). Of note, cholinergic neurons of the striatum are relevant in the context of ciliogenesis, as they are involved in a neuroprotective signalling circuit with dopaminergic neurons where their cilia are required to sense a sonic hedgehog signal transmitted by dopaminergic neurons (Caspary et al., 2007). Due to the importance of centrosome and cilia function for cellular health, the function of LRRK2 in ciliogenesis and centrosome cohesion has also been proposed as a major contributor to PD pathogenesis (Fasiczka et al., 2023).

In addition to an effect on endolysosomal and centrosomal functions, LRRK2 has been linked to impaired mitochondrial health and concomitant inflammatory signalling (Cherra et al., 2013; Wauters et al., 2020; Weindel et al., 2020). The involvement of LRRK2 in mitochondrial fusion and fission, as well as in mitophagy, has been proposed (for a recent review, see Singh and Ganley, 2021). For instance, it has been suggested that LRRK2 kinase activity might reduce PINK1–Parkin-mediated mitophagy, and that G2019S substitution in LRKK2 could exacerbate this impairment (Bonello et al., 2019; Wauters et al., 2020). In a similar vein, an inverse correlation has been reported between LRRK2 kinase activity and basal mitophagy levels, implying that the LRRK2 G2019S gain-of-function mutation exerts its effect by impairing mitochondrial homeostasis (Singh et al., 2021).

Whether directly or indirectly, LRRK2 has been suggested to play a role in many other cellular processes, including cytoskeletal dynamics (for a review, see Civiero et al., 2018), with evidence of LRRK2 interacting with microtubules (Gandhi et al., 2009) and actin (Meixner et al., 2011). Moreover, LRRK2 has been reported to be involved in organelle maintenance, Ca^2+^ (Gómez-Suaga and Hilfiker, 2012) and neuronal homeostasis, neurite outgrowth (Winner et al., 2011), and neurotransmitter release (Volta et al., 2017; Skiteva et al., 2022).

LRRK2 has also been implicated in the innate immune system, where it is highly expressed in innate immune cells such as monocytes, macrophages, neutrophils and B cells (Ahmadi Rastegar and Dzamko, 2020). Thus, it has been speculated that LRRK2 could play a role in clearance of and defence against intracellular pathogens (Härtlova et al., 2018; Ahmadi Rastegar et al., 2022). Furthermore, LRRK2 has been associated with inflammation through links to Dectin-1 (also known as CLEC7A) signalling, which is known to play a role in innate immunity against fungal infections (Drummond et al., 2011; Takagawa et al., 2018), and inflammasome activation (Liu et al., 2017), as well as diseases that are thought to be caused by immune dysregulation, such as leprosy and Crohn's disease (Shutinoski et al., 2019).

Unsurprisingly, these LRRK2-regulated processes could be connected, as seems to be the case for the impact of LRRK2 on mitochondrial homeostasis, which subsequently results in the dysregulation of the innate immune system (Weindel et al., 2020). Further investigation and resolution of potential physiological substrates for LRRK2 is a priority, as this would allow a better understanding of the interacting partners, protein networks and cellular roles of this multifaceted enzyme.

## LRRK2 – dysfunction and disease

### LRRK2 and human disease genetics

As noted above, autosomal-dominant missense mutations in the *LRRK2* gene are associated with familial PD (Paisán-Ruiz et al., 2004; Zimprich et al., 2004). Mutations in *LRRK2* are one of the most prevalent genetic causes of PD, with more recent data from genome-wide association studies identifying common variants at the *LRRK2* locus on chromosome 12 as being linked to increased risk of idiopathic disease (Nalls et al., 2019). Of note, the disease-associated mutations are all found within the enzymatic core of LRRK2, with common mutations found in the ROC domain (N1437H/D/S and R1441C/G/H), the COR domain (Y1669C) or in the kinase domain (G2019S and I2020T) (Cookson, 2010) (see poster). Two variants in particular, N1437D (in East Asian populations) and G2019S (primarily in North African subpopulations), are the most common mutations identified in *LRRK2* (Lesage et al., 2006; Zhao et al., 2020; Simpson et al., 2022).

Mechanistically, how LRRK2 is implicated with PD pathology remains uncertain. At biochemical and cellular levels, there is extensive evidence to support dysregulation of LRRK2 enzymatic activity as a key event in PD, notably with coding mutations in the *LRRK2* gene resulting in increased kinase activity (Alessi and Sammler, 2018). How these changes in LRRK2 kinase activity lead to neurodegeneration is less clear, with a wide range of cellular pathways reported as being altered in cellular and animal models of disease. Distinct consequences suggested as a result of this enhanced enzymatic activity include increased aggregation and spreading of the PD-associated proteins α-synuclein (encoded by *SNCA*; Bae et al., 2018) and/or tau (encoded by *MAPT*; Bailey et al., 2013; Herbst et al., 2022), overactivation of microglia and concomitant neuroinflammation (Moehle et al., 2012), dysregulation of downstream signalling pathways, and altered intracellular trafficking following hyperphosphorylation of Rab proteins (Madero-Pérez et al., 2018; Sobu et al., 2021; for a recent review, see Boecker, 2023). These are all plausible routes to neurodegeneration; however, further work is required to understand which of them predominate. Intriguingly, evidence is accruing to support a pathogenic interplay between *LRRK2* and other genes with established links to PD, including *PINK1*, *PRKN*, *VPS35*, *GBA1*, *SNCA* and *MAPT* (reviewed in Cookson, 2015; Cresto et al., 2019).

Beyond PD, genome-wide association studies have identified *LRRK2* as a disease risk locus in a number of other diseases, including leprosy (Zhang et al., 2009) and inflammatory bowel disease (Franke et al., 2010). Most recently, variants at the *LRRK2* locus have been linked to the rate of progression in progressive supranuclear palsy (PSP), a parkinsonian disorder with some clinical overlap with PD, but a distinct aetiology (Jabbari et al., 2021). In addition to these, LRRK2 could play a role in cancer, given the identification of *LRRK2* variants associated with different types of carcinomas (Agalliu et al., 2015; Ermine et al., 2022; Saunders-Pullman et al., 2010). However, discrepancies have been observed across studies (Ruiz-Martínez et al., 2014), emphasising that the mechanistic involvement of the *LRRK2* gene in cancer remains enigmatic.

### Targeting LRRK2

The prominence of *LRRK2* in the genetic landscape of PD has resulted in widespread efforts to target and modulate LRRK2 activity, with the goal of modifying the progression of PD. Kinase inhibitors specific to LRRK2 have been developed by both academic researchers and industrial groups, including MLi2, PFE360 and GNE0877 (Baptista et al., 2020; Estrada et al., 2014; Fell et al., 2015). Several inhibitors have undergone phase I and phase II clinical trials (Lewis, 2022), and a small-molecule LRRK2 kinase inhibitor developed by Denali Therapeutics and Biogen (DNL151/BIIB122, https://clinicaltrials.gov/ct2/show/NCT05418673) has progressed through to phase III efficacy trials for use in PD. Other therapeutic approaches, including modulating GTPase activity (Li et al., 2021) and targeting LRRK2 with antisense oligonucleotides (Zhao et al., 2017), have also been developed. The latter approach is now undergoing phase I clinical trials to assess safety, led by Ionis Pharmaceuticals and Biogen (designated BIIB094, https://www.clinicaltrials.gov/ct2/show/NCT03976349). Subject to the outcome of these trials, a key question is whether targeting LRRK2 will be beneficial to those living with PD but without a mutation in *LRRK2*, or for disorders beyond PD that involve LRRK2.

## Conclusions

The complexities of the biology and genetics of LRRK2 make it a fascinating and challenging subject for investigation. The past few decades since the first functional analyses of LRRK2 have witnessed substantial advances in our understanding of the genetics, structure, function and role of this protein. Even in the past five years, the elucidation of multiple structures for LRRK2 and the characterisation of signal transduction events regulated by LRRK2 (in particular, phosphorylation of the Rab proteins) have substantially expanded the horizons for LRRK2 research. As is clear from the discussions above, there remain significant gaps in our understanding of LRRK2 biology and pathology. Given the interest in this protein, both as a drug target for human disease and as a multifaceted regulator of cellular events, it is likely that further pieces of the LRRK2 puzzle will fall into place in the coming years, which promise to be exciting for the field of LRRK2 research.

## Poster

Poster

## Panel 1. Structure and interactome

Panel 1. Structure and interactome

## Panel 2. Cellular functions

Panel 2. Cellular functions

## Panel 3. Signal transduction

Panel 3. Signal transduction

## Panel 4. Human diseases associated with LRRK2

Panel 4. Human diseases associated with LRRK2
