# Supplementary figures and images for: Leucine-rich repeat kinase 2 at a glance
Source: J Cell Sci. 2023 Sep 12;136(17):jcs259724. doi: 10.1242/jcs.259724 (PMC10508695; doi:10.1242/jcs.259724)

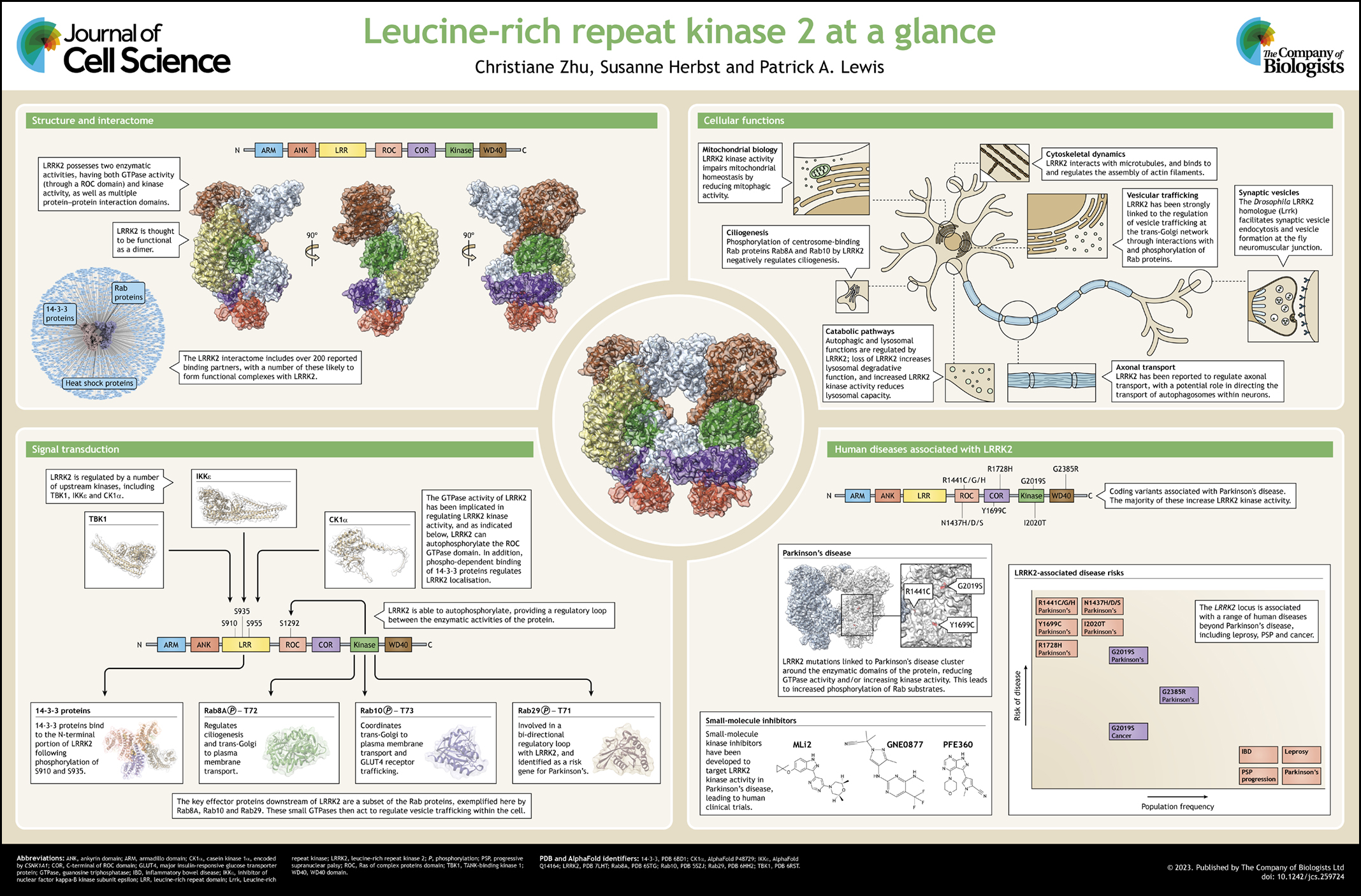

Supplement: Poster [file joces-136-259724-s1.jpg]

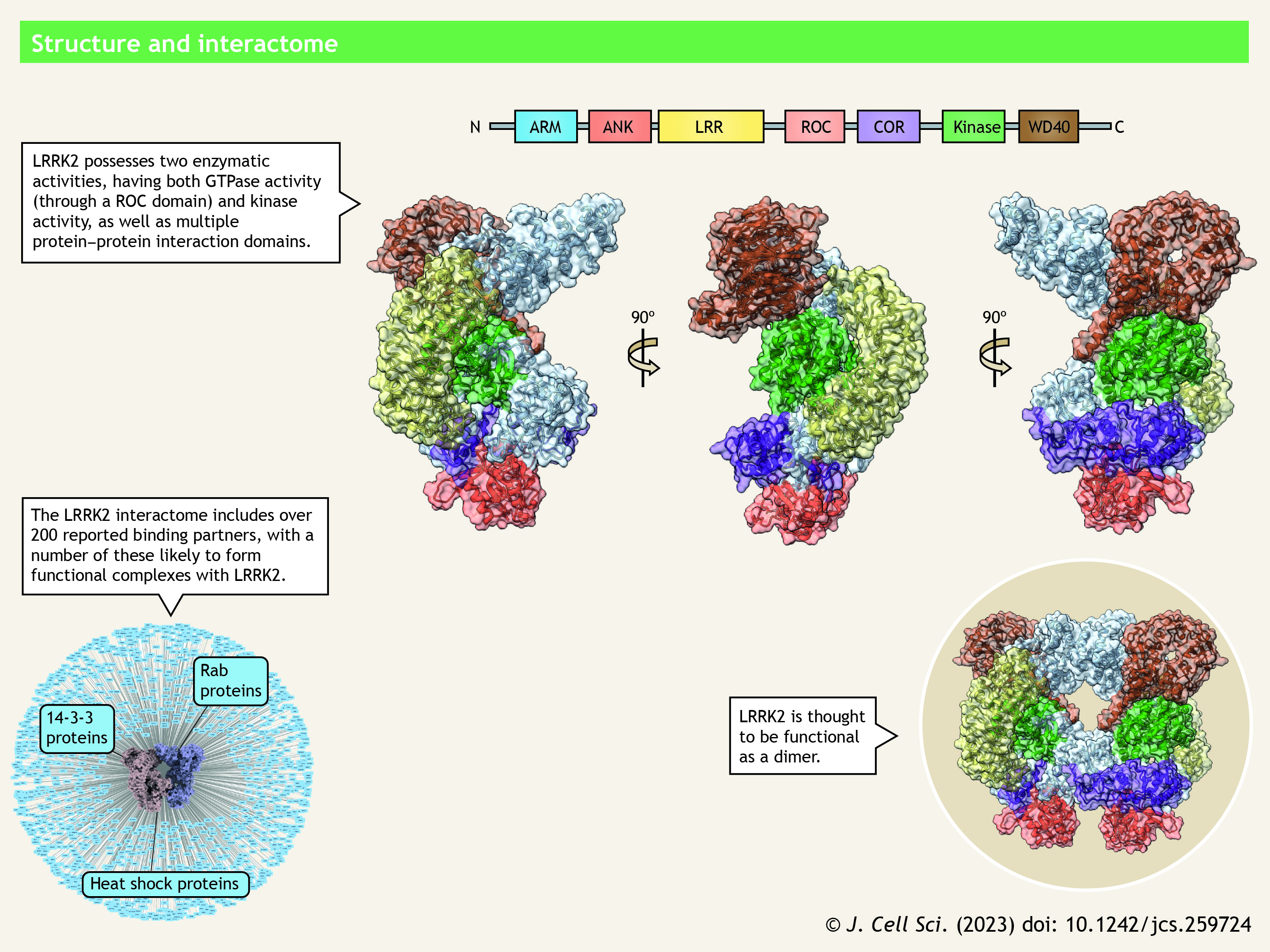

Supplement: Panel 1. Structure and interactome [file joces-136-259724-s2.jpg]

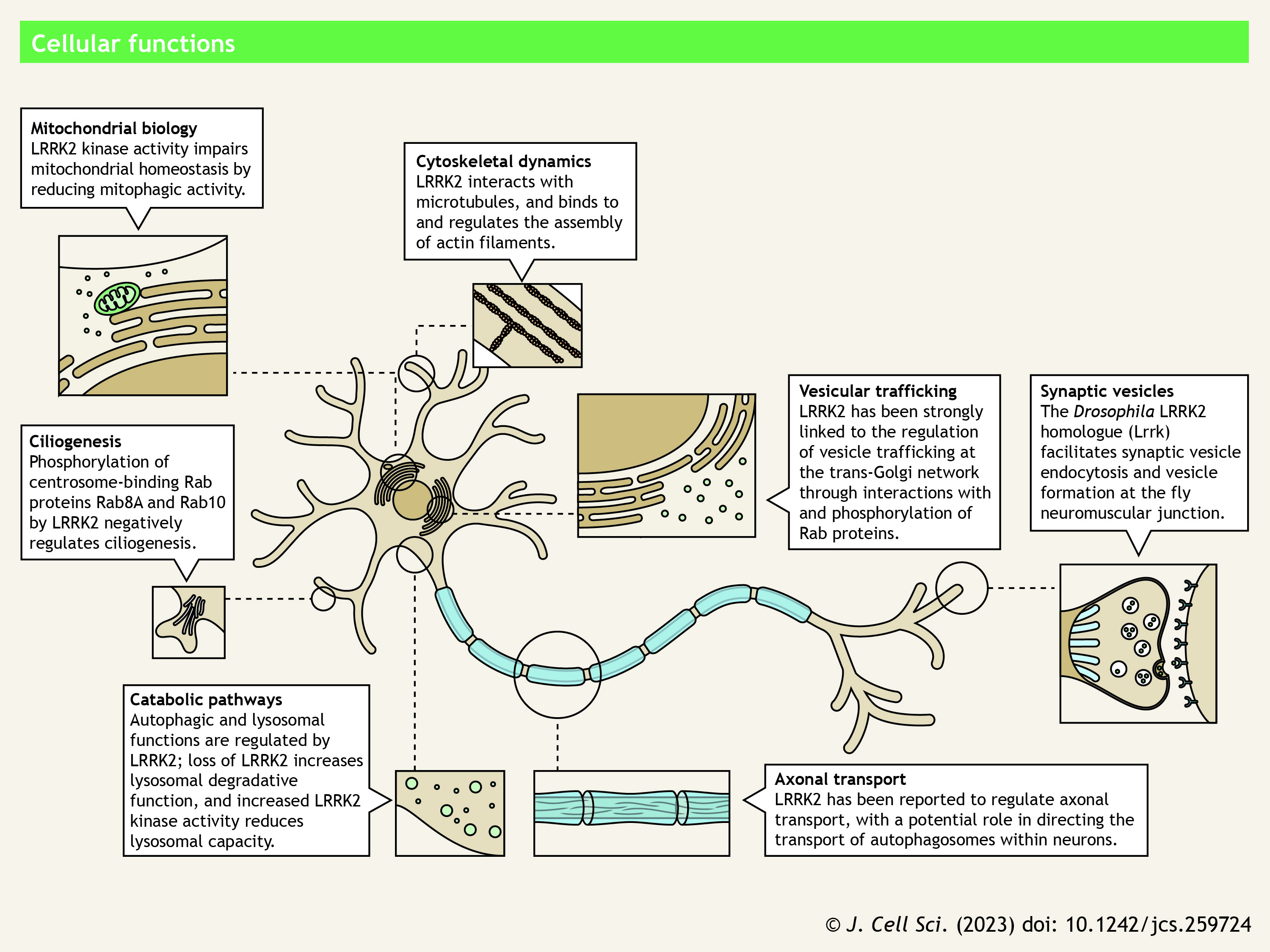

Supplement: Panel 2. Cellular functions [file joces-136-259724-s3.jpg]

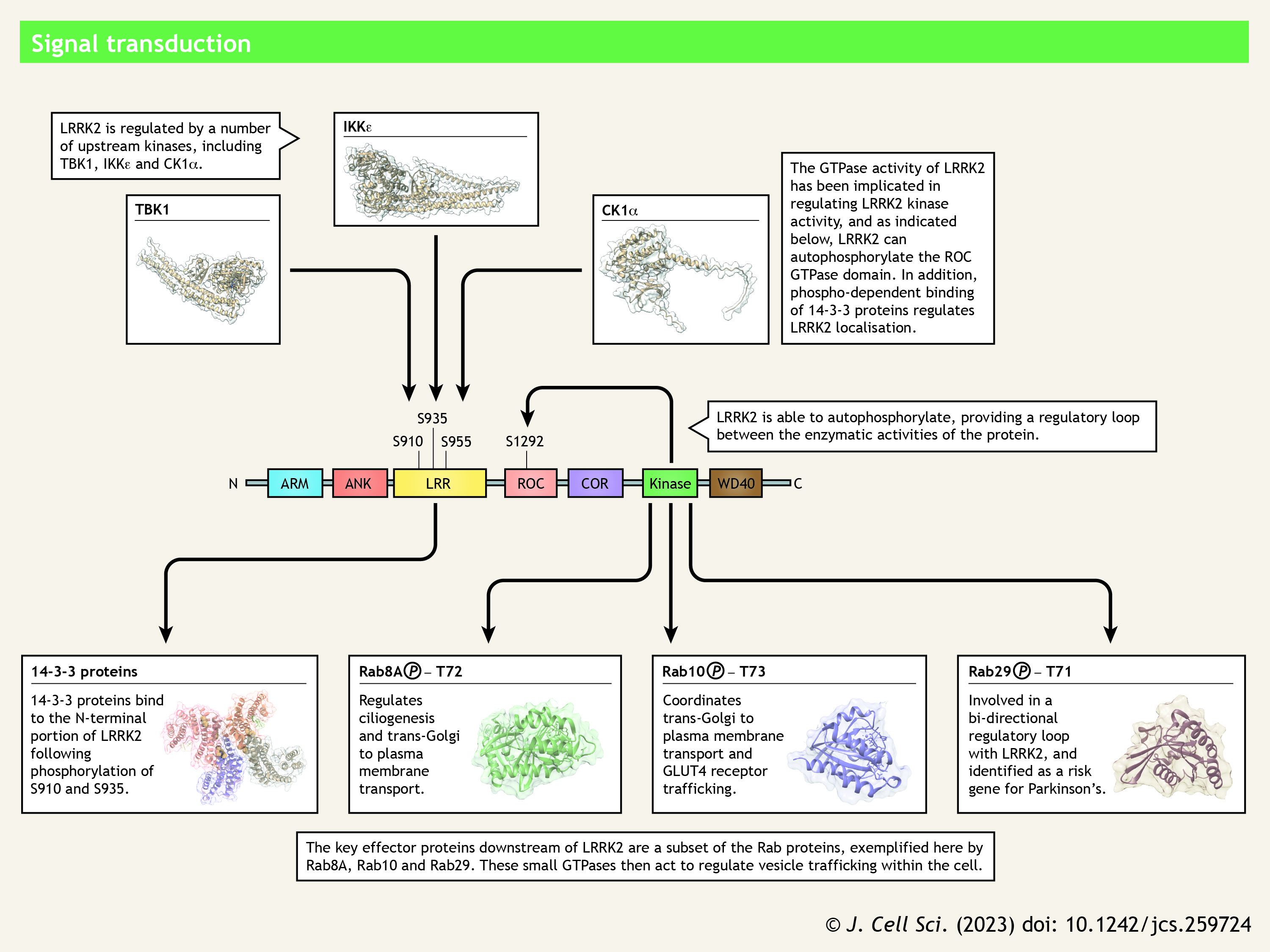

Supplement: Panel 3. Signal transduction [file joces-136-259724-s4.jpg]

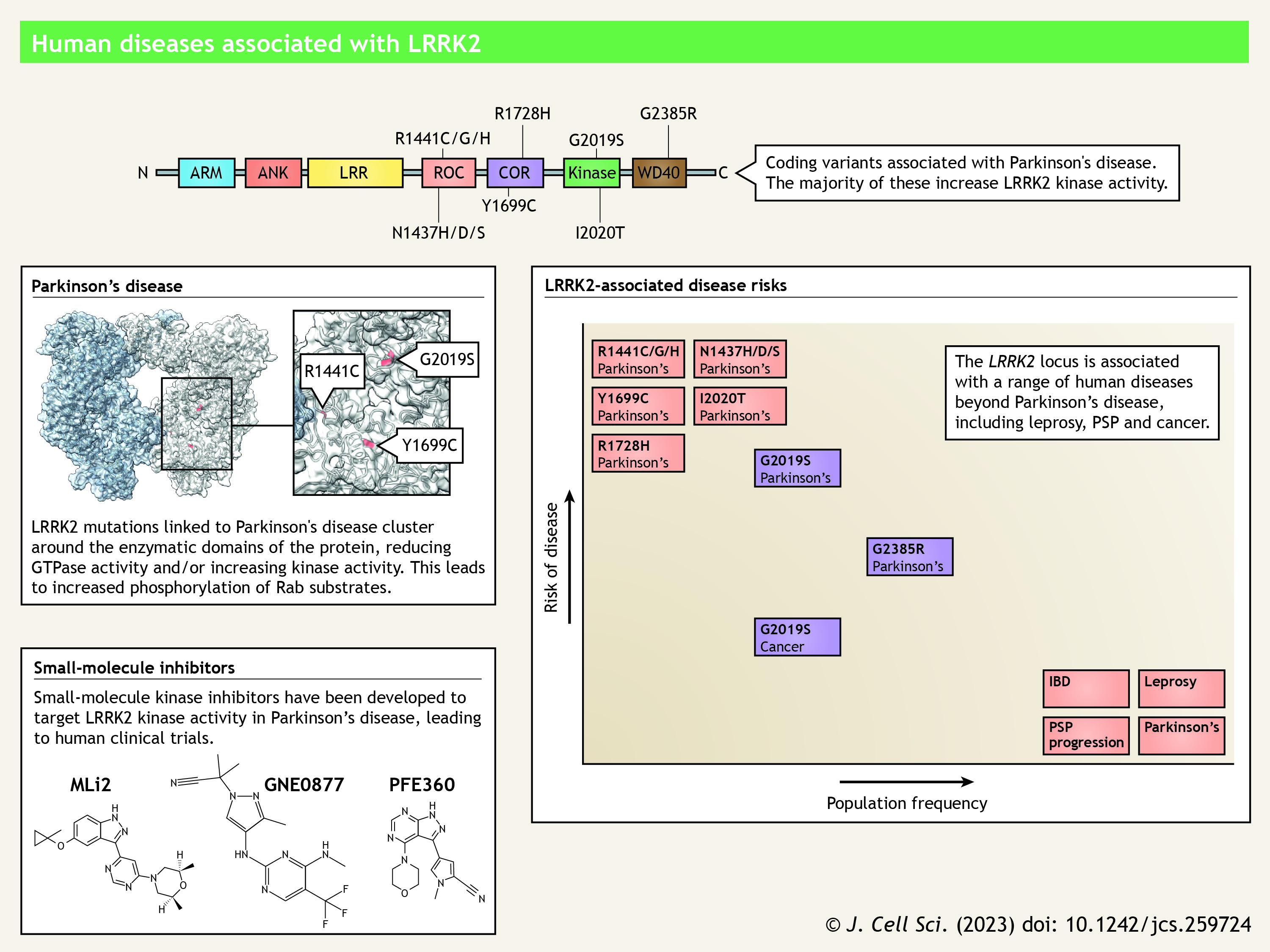

Supplement: Panel 4. Human diseases associated with LRRK2 [file joces-136-259724-s5.jpg]
